# Natural Bioactive Compounds in Dental Materials: Balancing Biological Activity and Functional Properties

**DOI:** 10.3390/pharmaceutics18040462

**Published:** 2026-04-09

**Authors:** Dana Gabriela Budala, Ionut Luchian, Teona Anamaria Tudorici, Andrei Georgescu, Florinel Cosmin Bida, Oana Cioanca, Nicoleta Tofan, Ancuta Goriuc, Gabriel Rotundu, Monica Hancianu

**Affiliations:** Grigore T. Popa University of Medicine and Pharmacy, 700115 Iasi, Romania

**Keywords:** bioactive natural compounds, dental materials, bioactive coatings, surface functionalization, plant-derived compounds

## Abstract

The integration of bioactive natural compounds into dental materials has gained increasing attention as a strategy to improve biological functionality while maintaining material performance. This narrative review aims to synthesize current evidence regarding the main classes of natural compounds investigated in dental materials, their incorporation methods, and their influence on material properties. A literature-based narrative approach was conducted using major scientific databases, including PubMed, Scopus, and Web of Science, focusing on studies addressing natural compound incorporation into restorative, prosthetic, adhesive, cementitious, and hydrogel-based dental materials. The reviewed literature indicates that polyphenols, polysaccharides, proteins and peptides, terpenoids, and microbial- and marine-derived compounds have been incorporated using bulk modification, surface functionalization, coating systems, and hybrid material architectures. While these compounds may provide antimicrobial, antioxidant, and bioactive properties, they may also influence mechanical behavior, physicochemical stability, optical characteristics, surface properties, and release behavior, depending on compound chemistry, concentration, and incorporation strategy. The available evidence highlights the need for a balanced approach that considers both biological activity and material performance, as well as the importance of stability, standardization, and long-term clinical performance when integrating natural bioactive compounds into dental materials.

## 1. Introduction

Natural materials and plant-derived compounds have been used in dentistry since early therapeutic practices, particularly in the form of phytochemicals applied for oral care and restorative purposes [1]. Herbal extracts, resins, and naturally occurring minerals have long been utilized for their antibacterial and calming effects in the treatment of oral diseases [2,3]. With the development of modern dental materials, naturally derived bioactive compounds continued to be explored as functional additives, initially in preventive formulations and later in restorative and prosthetic applications [4,5,6].

Early research focused primarily on their antibacterial and anti-inflammatory effects in contact with oral tissues, which laid the groundwork for the contemporary interest in incorporating naturally derived bioactive into dental material systems [7]. These bioactive compounds used in dental materials encompass a structurally diverse group of molecules, including polyphenols, flavonoids, polysaccharides, and naturally derived macromolecules [8].

Advances in analytical techniques enabled a clearer understanding of their chemical composition, stability, and variability between natural sources, while improvements in extraction, purification, and standardization allowed more consistent evaluation of their behavior within dental material systems [9,10]. Their chemical structure includes functional groups responsible for antibacterial, antioxidant, and anti-inflammatory properties, while structural characteristics such as molecular weight, polarity, and functionalization degree influence their stability and compatibility in material systems [11,12]. These characteristics determine how natural compounds interact with polymer matrices, ceramic surfaces, or interfacial layers, making structural understanding essential before evaluating their influence on material behavior [13,14].

In addition to their biological effects, biologically active substances generated from natural sources interact with matrices of dental materials through a number of physicochemical pathways. The bioactive molecules’ functional groups and the matrix’s chemical structure determine the type of interactions that may occur, which can range from hydrogen bonding and van der Waals interactions to ionic and covalent bonding [12]. The behavior of polymerization, the density of cross-linking, and the network structure can all be impacted by natural substances based on materials based on polymers; on the other hand, surface energy and interfacial adhesion can be affected by ceramic or inorganic matrices [12,13]. Mechanical strength, water sorption, degradation behavior, controlled release profiles, and other material attributes can be affected by the addition of natural chemicals. Incorporating natural bioactive chemicals into dental material design and performance prediction requires an understanding of these mechanistic connections [14].

**Figure 1:**
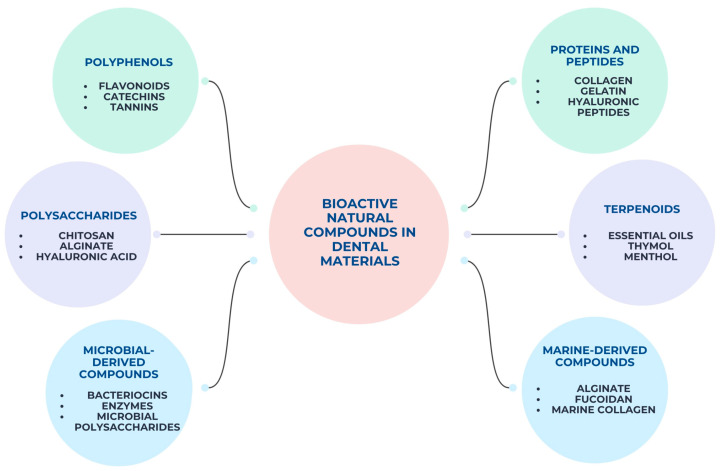
Schematic overview of the main classes of bioactive natural compounds.

As natural compounds became better characterized and more consistently integrated into dental materials, the scientific literature expanded rapidly and in a fragmented manner across different material types and applications. Studies addressing natural compounds have been dispersed among diverse dental material categories, often focusing on specific formulations or isolated effects [15,16]. To provide a structured overview of the main classes of bioactive natural compounds investigated in dental materials, Figure 1 schematically illustrates the principal compound categories, together with representative examples commonly reported in literature.

This dispersion has made it difficult to obtain a coherent view of how natural compounds are incorporated within material systems. Consequently, a more structured synthesis of existing evidence is required to clarify their role in contemporary dental materials.

Despite the increasing number of studies investigating naturally derived bioactive compounds in dental materials, several important questions remain unresolved. There is still limited consensus regarding optimal extraction and standardization methods, long-term stability of bioactive compounds within dental material matrices, controlled release behavior, and their influence on mechanical and physical properties of dental materials. Furthermore, comparisons between different classes of natural compounds and their effectiveness across various dental material systems remain insufficiently clarified.

From a translational point of view, adding natural bioactive compounds to dental materials may help them work better with biological systems and interact better with oral tissues. These materials may help soft tissues heal, lower the number of bacteria that grow at restoration margins, make restorative interfaces last longer, and improve long-term clinical outcomes. Thus, it is essential to understand the way organic chemicals and dental material matrix function when combined. The result has implications for both material science as well as developing bioactive dental materials that individuals may employ in daily life.

By bringing together studies from different material categories and research contexts, this review seeks to present an overall picture of how natural compounds have been explored within dental material research and to identify current research trends, reported challenges, and directions for future research.

## 2. Literature Review

The methodological approach used in this review was tailored to the heterogeneous nature of the available literature on bioactive natural compounds in dental materials. A narrative review framework was therefore adopted to allow a flexible synthesis of studies differing in material systems and research focus.

✔ Study Design

This study was designed as a narrative review to provide a comprehensive and structured overview of the existing literature on natural bioactive compounds incorporated into dental materials. This approach was selected because the topic encompasses a broad range of material types, compound classes, and application methods, making a strictly systematic comparison difficult [17].

✔ Literature Sources

The literature search was performed using major scientific databases relevant to dental materials and biomaterials research, including PubMed, Scopus, and Web of Science. These databases were selected to ensure broad coverage of peer-reviewed studies published in dental, science materials, and interdisciplinary journals. The following keywords and Boolean operators were used: (“natural compounds” OR “plant-derived compounds” OR “natural extracts” OR “bioactive agents” OR “phytochemicals”) AND (“dental materials” OR “restorative materials” OR “dental polymers” OR “resin composites” OR “ceramics” OR “dental cements” OR “adhesives” OR “surface coatings”).

Manual cross-referencing of cited literature within key publications was performed to ensure inclusion of seminal studies and relevant experimental reports. Reference lists of relevant articles were manually screened to identify additional publications of interest. The search included peer-reviewed articles published in English between January 2000 and December 2025.

✔ Study Selection

Studies were included if they investigated the incorporation of bioactive natural compounds into dental material systems, with a clear focus on material formulation, modification, or characterization. Eligible studies encompassed in vitro, experimental, and material-oriented research addressing naturally derived compounds integrated into restorative, prosthetic, or related dental materials. Both synthetic–natural hybrid systems and surface-modified materials were considered.

Studies were excluded if they focused solely on clinical performance without material-level analysis, addressed non-dental applications, or investigated natural compounds used exclusively as therapeutic agents without incorporation into a material matrix. Reviews, conference abstracts, and non–peer-reviewed publications were not considered.

Only peer-reviewed articles published in English within the defined time frame were considered. The selection process was guided by thematic relevance and consistency with the scope of the review, without applying formal quantitative quality assessment or risk-of-bias evaluation.

In addition to the general inclusion criteria, priority was given to studies that investigated the incorporation of natural bioactive compounds into dental materials, their influence on physicochemical, mechanical, optical, and biological properties, as well as studies addressing clinical relevance and potential dental applications. Recent publications were prioritized in order to reflect current research trends, while review articles were used to provide a broader scientific context. Experimental studies with clear methodology and relevance to dental material performance were considered particularly important for the present review.

✔ Data Extraction and Synthesis

**Figure 2:**
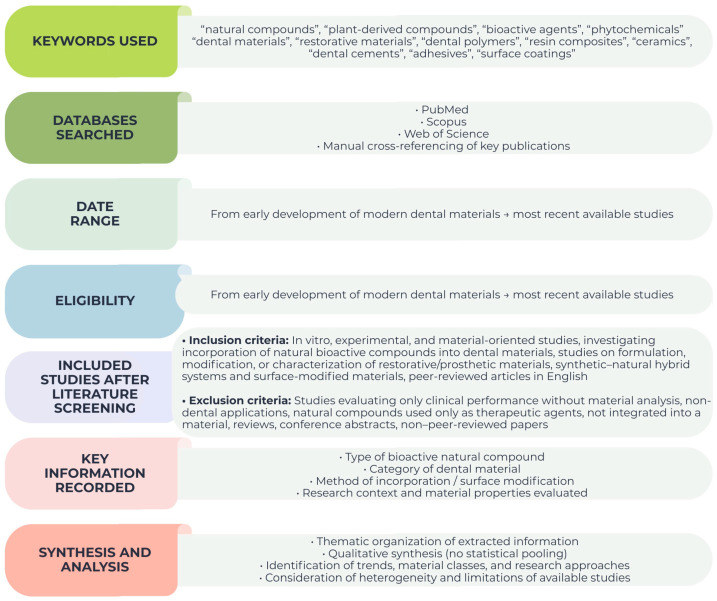
Overview of the literature search strategy, eligibility criteria, and data synthesis approach used in this narrative review.

Relevant information was extracted from the included studies focusing on aspects directly related to the scope of the review. Extracted data included the type of bioactive natural compound investigated, the category of dental material involved, and the general approach used for compound incorporation. In addition, information regarding the reported effects on their properties. was also collected when available. Particular attention was given to the methods of incorporation of natural compounds into dental materials, including bulk incorporation, surface functionalization, coatings, and hybrid natural–synthetic systems. When reported, information regarding release behavior, stability of the incorporated compounds, and interactions between the natural compounds and the material matrix was also considered. Additional information regarding the reported research context and material-related observations was also recorded when available and all information was synthesized in Figure 2:

The extracted data were analyzed qualitatively and grouped according to the type of natural compound, the dental material category, and the primary functional effects observed. The synthesis of the literature was performed in a narrative manner, aiming to identify major research directions, commonly used incorporation strategies, reported ad-vantages and limitations, as well as existing gaps in the current literature.

Research addressing the use of bioactive natural compounds in dental materials has expanded considerably over the past decades, reflecting growing interest in biologically responsive material systems. The available literature encompasses a wide variety of naturally derived substances investigated across different material platforms, often with differing experimental aims and levels of characterization.

### 2.1. Classes of Bioactive Natural Compounds Investigated in Dental Materials

Studies exploring natural compounds in dental materials describe a chemically diverse group of substances derived from plant, microbial, and animal sources. Rather than representing a uniform category, these compounds differ markedly in molecular structure, origin, and functional attributes, a diversity that has shaped the breadth of material systems and research strategies reported in the literature [18,19].

For thousands of years, people all over the world have turned to medicinal herbs as a means of relieving a wide range of health problems. About a quarter of all medications come from plants or plant extracts, and traditional medicine is the main method of treatment in rural areas of poor countries [20,21]. Herbal treatments are relied upon by 80% of the world’s population for basic healthcare needs because they are culturally acceptable, inexpensive, and easy to get, according to the World Health Organization [22].

Recent research has shown that traditional medicine has a significant role in the treatment of oral disorders [23]. Theoretically, medicinal herbs also include volatile oils that promote blood flow, tannins that clean and tighten gum tissue, and other ingredients like vitamin C that keep gums healthy [24]. So yet, only a small number of plants have been granted approval for their valuable therapeutic characteristics, and little is known about the toxicity of these plant-based medications because no randomized controlled clinical research has been conducted [25].

#### 2.1.1. Polyphenols and Flavonoids

**Figure 3:**
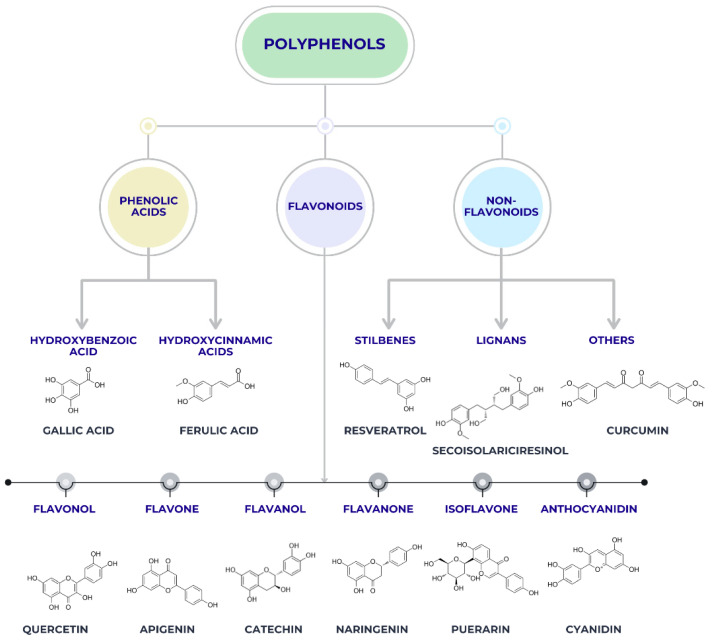
Classification of polyphenols into phenolic acids, flavonoids, and non-flavonoid compounds, with representative molecules relevant for dental material research.

Plant-derived compounds, particularly polyphenols, are among the most frequently investigated natural agents in dental material research [26]. Within this broad group, flavonoids such as catechins, quercetin, curcumin, and resveratrol represent a structurally distinct subclass that has attracted considerable attention [27]. These molecules exhibit antibacterial, antioxidant, and anti-inflammatory properties. From a materials perspective, polyphenols have attracted interest due to their ability to interact with polymeric matrices via hydrogen bonding and π–π interactions, potentially influencing polymer crosslinking density and surface chemistry [28]. Several studies have demonstrated that the incorporation of flavonoids into resin-based composites, adhesives, or coating systems can impart antimicrobial functionality without significantly compromising mechanical performance when used at optimized concentrations [29]. However, issues related to color stability, photodegradation, and long-term release kinetics remain important challenges for their clinical translation [30]. Figure 3 presents the main classification of polyphenols into phenolic acids, flavonoids, and non-flavonoid compounds. Phenolic acids include hydroxybenzoic and hydroxycinnamic acids, while flavonoids include several subclasses such as flavanols, flavones, flavanols, flavanones, isoflavones, and anthocyanidins. Non-flavonoid polyphenols include stilbenes, lignans, and other compounds such as curcumin. These compounds are frequently investigated in dental materials due to their antioxidants, antibacterial, and bioactive properties.

#### 2.1.2. Polysaccharides

Among the many natural substances studied for potential use as dental materials, polysaccharides stand out [31]. Biocompatibility and surface/interface modification capabilities of naturally occurring macromolecules like chitosan, alginates, and cellulose derivatives have been investigated [32]. Rather than being mentioned as main structural components, polysaccharides are often characterized as functional modifiers that impact material behavior [33].

Chitosan, in particular, has been widely explored due to its cationic nature, which enables electrostatic interactions with negatively charged bacterial cell membranes and dental hard tissues [34]. There is no universally accepted cutoff for chitosan degree of deacetylation (DD%), although research shows that lysozyme-mediated breakdown of glycosidic linkages occurs at a significantly higher rate in chitosan with a DD% of 70–75% [35]. The polymer becomes more cationic and enzymatically sensitive when the DD% approaches 85%, leading to rapid resorption, which has even more noticeable effects [35]. Quicker bio absorption is typically associated with greater DD% values; however, the exact degradation rate is affected by factors such as molecular weight, crystallinity, and the degree of crosslinking in the final substance [22,35].

The effects of a chitosan-collagen sponge on periodontal tissue regeneration in beagle dogs with surgically induced one-wall bone defects were examined in an eight-week preclinical investigation by Park et al. After eight weeks, histological and histometric investigations showed that the chitosan group had much less apical migration of the junctional epithelium, which is a good thing because it helps with periodontal regeneration. Newly produced cementum and alveolar bone were also significantly higher in this group than in the control and buffer groups [36].

For the purpose of controlling bleeding, especially during bone surgery, Al-Mofty et al. created multifunctional haemostatic sponges made of chitosan and poly (vinyl alcohol) that are enhanced with hydroxyapatite and ciprofloxacin [37]. As a result of the synergy between ciprofloxacin and hydroxyapatite, the hybrid sponge exhibited the best qualities, including excellent mechanical strength, platelet aggregation capability, and high biocompatibility [37]. The sponges showed a remarkable potential to swell and degrade quickly, losing 70% of their bulk after only 7 days [37]. This sponge showed promise as a surgical instrument for use in bone and tooth procedures, according to the study [37].

Several other dental uses of chitosan have been explored, alongside its antibacterial and film-forming capabilities. Remineralization studies have focused on chitosan-based systems because of their capacity to bind phosphate and calcium ions and encourage mineral deposition on surfaces of demineralized enamel [38]. The regulated release of antibacterial medicines, anti-inflammatory medications, or growth factors in the oral environment can be achieved through the extensive exploration of chitosan as a carrier in drug delivery systems [39]. Periodontal dressings and regenerative applications have also made use of chitosan-based materials because of their biocompatibility, biodegradability, and capacity to promote tissue repair [40]. These new uses for chitosan in dentistry demonstrate the material’s adaptability and versatility.

In material systems, polysaccharides are commonly employed as reinforcing agents, surface modifiers, or components of hydrogel-based matrices [41]. Their hydrophilicity and molecular weight, however, may affect water sorption, dimensional stability, and long-term mechanical integrity, necessitating careful formulation and crosslinking strategies [42].

#### 2.1.3. Proteins, Peptides, and Protein-Derived Molecules

Proteins and peptides, including collagen, gelatin, antimicrobial peptides, and enamel matrix derivatives, represent a biologically inspired class of natural compounds with high relevance for dental materials [43]. These molecules are particularly attractive for applications targeting tissue integration, regenerative interfaces, and biomimetic surface design [44].

A peptide is a brief polymer made up of amino acids. Over the past few decades, researchers have examined over seven thousand native peptides (NP) that play crucial roles in human physiological processes. These NPs include cell adhesion motifs, structural peptides, peptide hormones, growth factors, matrix metalloprotease substrates, neuropeptides, peptide tags, amyloid peptides, and various others [45].

A number of studies have shown that peptides made from proteins found in enamel and dentin can slow or stop demineralization [46,47,48]. As an example, mature enamel lacks protein amelogenin, which is crucial for the development and proliferation of enamel crystals within the freshly created enamel matrix [49,50].

Researchers have shown that matrix metalloproteinase 20 proteolyzes amelogenin during biomimetic enamel regrowth [51]. This process results in a newly grown enamel-like layer that has improved mechanical properties, composition, structure, and well-regulated crystal growth [52,53].

Studies examining biological interactions at material-tissue interfaces also include reference to collagen-based materials and naturally generated proteins and peptides [54]. Composites and surface-modified systems frequently include these compounds due to the interest in their affinity for biological tissues and their possible involvement in biomaterial integration [55]. The biomedical sector has shown significant interest in collagen-derived materials due to their inherent features and adaptability. The pliability of collagen and the strength of hydroxyapatite come together in bone-like materials called collagen-hydroxyapatite composites (HA) [56]. These composites have great potential as implant coatings, scaffolds for bone regeneration, and bone grafts, among other uses in bone repair [57]. Collagen enhances cell adhesion, proliferation, and remodeling; HA boosts composite strength, promotes better bone contact, and stimulates bone formation [58,59,60].

Nevertheless, their susceptibility to enzymatic degradation, thermal instability, and potential immunogenicity pose significant limitations. As a result, protein-derived molecules are often used in modified or immobilized forms to improve their stability within synthetic material frameworks [61].

#### 2.1.4. Terpenoids and Essential Oil Components

Terpenoids and essential oil constituents, such as thymol, eugenol, carvacrol, and menthol, have long-standing use in dentistry due to their antimicrobial and anti-inflammatory properties [62]. In contemporary material science, these compounds are primarily investigated as functional additives in sealers, liners, temporary materials, and surface coatings [63].

Many aromatic plants’ essential oils contain monoterpenes, a type of terpenoids composed of two isoprene units [64], which are present in thyme, peppermint, eucalyptus, and many more [65]. Their antibacterial, anti-inflammatory, and antioxidant capabilities are only a few of the biological actions they display. Antibacterial activities of important monoterpenes including thymol and carvacrol have been investigated in detail, and results show that they are effective against both Gram-positive and Gram-negative bacteria [66]. These medicines are highly effective against bacterial infections because of their varied modes of action, which often involve disrupting the bacterial cell membrane, interfering with enzyme activity, and inhibiting biofilm development. In addition, thymol causes cell death via increasing ROS buildup and DNA damage through intercalation while carvacrol can amplify the inhibitory effects of certain conventional drugs, including tetracycline, erythromycin, and fluconazole [67].

While their antimicrobial efficacy is well documented, their high volatility and potential plasticizing effects on polymer networks require precise control of concentration and delivery. Encapsulation strategies and controlled-release systems have therefore been proposed to mitigate rapid diffusion and maintain material stability [68].

Despite their less common occurrence, these compounds show how research is still in its exploratory stages and how the types of natural compounds studied in dental materials are constantly growing [69].

#### 2.1.5. Microbial- and Marine-Derived Natural Compounds

An emerging area of interest involves bioactive compounds derived from microbial and marine sources, including bacteriocins, bioactive peptides, and polysaccharides isolated from algae or marine organisms [70]. These substances offer novel antimicrobial mechanisms and unique physicochemical properties that may complement conventional dental materials [71]. Unlike conventional plant-derived molecules, these natural agents originate from highly adaptive biological systems exposed to extreme environmental conditions, which confer distinctive structural and functional properties.

Bacteriocins, ribosomal synthesized antimicrobial peptides produced by bacteria, have gained attention for their targeted antibacterial activity against oral pathogens. Unlike broad-spectrum antibiotics, bacteriocins often exhibit selective activity, reducing pathogenic biofilm formation while preserving beneficial commensal microbiota [70,71]. This specificity is particularly advantageous in the oral cavity, where ecological balance plays a critical role in maintaining health. Moreover, certain bacteriocins demonstrate anti-biofilm properties by disrupting quorum sensing pathways and interfering with bacterial adhesion, making them promising candidates for incorporation into restorative materials, sealants, and periodontal dressings [72].

Marine-derived bioactive peptides represent another promising class of compounds. These molecules often display multifunctional properties, including antimicrobial, anti-inflammatory, antioxidant, and pro-regenerative effects [70]. Due to their amphiphilic structures, many marine peptides can interact with microbial membranes while simultaneously modulating host cell signaling pathways [71]. In the context of oral wound healing, such peptides may contribute to immune regulation, enhancement of fibroblast activity, and acceleration of tissue regeneration. Their relatively small size and structural versatility make them suitable for integration into hydrogel matrices and controlled-release biomaterial systems [71].

Marine-derived polysaccharides such as alginate, carrageenan, and chitosan derivatives have gained increasing attention in dental biomaterials due to their biocompatibility, gel-forming ability, and capacity to support drug delivery and tissue regeneration. Alginate is widely used in hydrogel systems and scaffolds due to its ability to form three-dimensional networks in the presence of calcium ions, making it suitable for tissue engineering and regenerative applications [71].

Carrageenan has been investigated for hydrogel formulations and controlled drug release systems due to its sulfated polysaccharide structure and gel-forming properties. Chitosan derivatives obtained from marine sources have also been studied for scaffold fabrication, drug-delivery matrices, and regenerative applications due to their biodegradability, antimicrobial properties, and ability to support cell adhesion and tissue healing [70,72]. These marine polysaccharides represent an important class of naturally derived biomaterials with potential applications in hydrogels, scaffolds, and drug-delivery systems used in dentistry.

**Figure 4:**
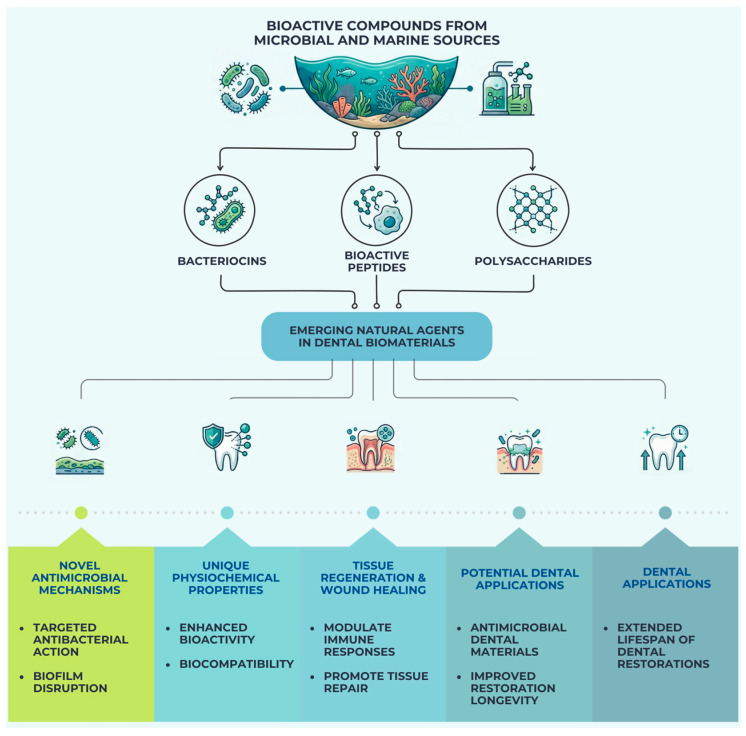
Bioactive compounds derived from microbial and marine sources and their potential applications in dental biomaterials, including antimicrobial mechanisms, physicochemical properties, tissue regeneration, and dental material applications.

Figure 4 illustrates the main categories of bioactive compounds derived from microbial and marine sources, including bacteriocins, bioactive peptides, and polysaccharides. These compounds are increasingly investigated as emerging natural agents in dental biomaterials due to their antimicrobial activity, biocompatibility, and regenerative potential.

In addition to polysaccharides such as chitosan, several other natural compounds have been extensively investigated for dental applications, including polyphenols, essential oils, propolis, and plant-derived flavonoids. These compounds have been studied for their biological activity and their potential incorporation into dental materials and preventive formulations.

Essential oils, for example, have been widely investigated in dentistry due to their therapeutic properties and antimicrobial activity, being commonly used in oral care products and dental formulations [73]. Polyphenols and flavonoids have been studied for their antioxidant and antibacterial properties, as well as for their ability to interact with dental tissues and biomaterials [74]. Propolis, a natural resinous product, has also been investigated for its antimicrobial and regenerative potential in dental applications [75]. The inclusion of these compounds highlights the diversity of natural bioactive substances investigated in dental material research.

#### 2.1.6. Nano-Based Systems Containing Natural Compounds

Nanotechnology has enabled new applications of natural bioactive compounds in dentistry by improving their stability, bioavailability, and antimicrobial efficiency. Natural compounds incorporated into nanoscale systems have been investigated for several dental applications, including antimicrobial materials, implant surface modifications, drug delivery systems, and periodontal regeneration [76].

Natural compound-loaded nanoparticles have also been explored for implant surface coatings, where they may provide antibacterial effects and improve tissue integration. Such nanostructured coatings can release bioactive molecules in a controlled manner, reducing the risk of peri-implant infections [77].

Another important application is represented by nano-based drug delivery systems used in periodontal therapy and endodontics. Nanoparticles and nanofibers containing natural compounds can deliver antimicrobial and anti-inflammatory agents directly to periodontal pockets or root canal systems [78].

Nanostructured hydrogels and scaffolds incorporating natural compounds have also been investigated for periodontal and tissue regeneration applications, where they may promote cell adhesion, proliferation, and tissue healing [79].

Although still at an early stage of investigation, such compounds highlight the expanding scope of natural bioactive agents beyond terrestrial plant-derived molecules and underscore the need for systematic evaluation of their material compatibility and long-term performance.

### 2.2. Modes of Incorporation of Natural Compounds into Dental Materials

The incorporation of natural compounds into dental materials represents a critical step in translating their intrinsic properties into functional material systems [68]. Unlike conventional additives, natural compounds often present challenges related to chemical stability, compatibility with synthetic matrices, and processing conditions. Consequently, multiple incorporation strategies have been explored to balance material performance with compositional integrity [80].

Natural bioactive compounds have been incorporated into various dental materials for different clinical applications. For example, natural compounds have been investigated as antimicrobial coatings for dental implants to reduce bacterial adhesion and biofilm formation on implant surfaces [75]. Bioactive hydrogels containing natural compounds have been studied for periodontal regeneration and soft tissue healing due to their ability to deliver bioactive molecules and support tissue regeneration [42]. In restorative dentistry, natural compounds have been incorporated into composite resins, adhesives, and cements as anti-biofilm additives to reduce bacterial colonization at restoration margins and improve the longevity of restorations [30]. These applications demonstrate the potential of natural bioactive compounds to improve the biological and functional performance of dental materials in clinical practice.

**Figure 5:**
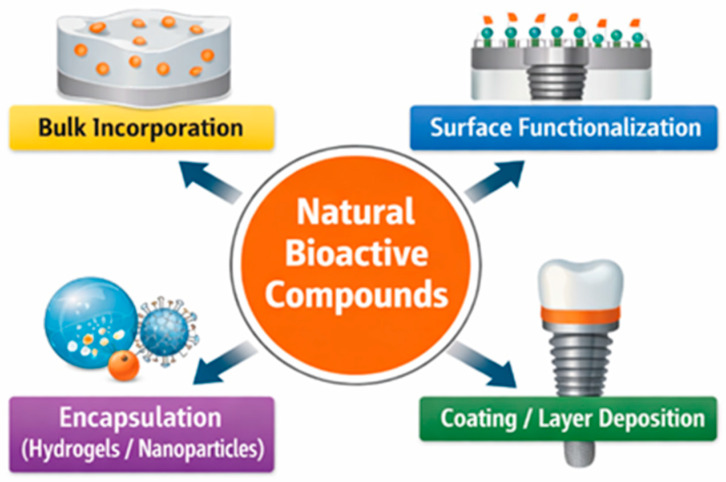
Main strategies for incorporating natural bioactive compounds into dental materials, including bulk incorporation, surface functionalization, coating or layer deposition, and encapsulation systems such as hydrogels and nanoparticles.

Natural bioactive compounds can be incorporated into dental materials through several strategies depending on the material type, compound chemistry, and desired release behavior. The main incorporation approaches include bulk modification, surface functionalization, coating or layer deposition, and encapsulation systems. These strategies are schematically illustrated in Figure 5.

#### 2.2.1. Bulk Incorporation Within the Material Matrix

Bulk incorporation involves the direct introduction of natural compounds into the bulk matrix of dental materials during formulation or fabrication, resulting in their uniform dispersion throughout the material volume. From a materials science standpoint, this strategy directly affects the polymer network architecture, phase behavior, and intermolecular interactions within the matrix [81].

In polymer-based systems, such as resin composites, adhesives, and resin-modified cements, bulk-added natural compounds may interact with monomers, oligomers, or polymer chains through hydrogen bonding, van der Waals forces, or π–π interactions, depending on their chemical structure [82]. These interactions can influence polymerization kinetics by altering radical mobility, initiation efficiency, or propagation rates, ultimately affecting the degree of conversion and crosslinking density [83]. Low-molecular-weight compounds may act as plasticizers, increasing chain mobility but potentially reducing stiffness and strength when presenting above critical concentrations [84].

Bulk incorporation can also impact network homogeneity and phase stability. Poor miscibility between natural compounds and the host matrix may lead to microphase separation, aggregation, or heterogeneity at the microscale, which in turn can act as stress concentrators and compromise mechanical performance. In contrast, compounds with compatible polarity or functional groups may integrate more effectively within the polymer network, preserving structural continuity [85].

Optical properties are particularly sensitive to bulk incorporation. Natural compounds with intrinsic chromophore structures may absorb visible or ultraviolet light, influence light transmission, translucency, and color stability, as well as potentially interfering with photo-initiated polymerization processes [86]. Such effects necessitate careful optimization of compound concentration and formulation, especially for light-cured restorative materials.

From a durability perspective, bulk incorporation may affect water sorption and aging behavior. Hydrophilic natural compounds can increase matrix affinity for water, promoting plasticization, hydrolytic degradation, or accelerated aging under oral-like conditions [87]. Additionally, leaching of poorly retained compounds may generate porosity or micro voids over time, further undermining mechanical integrity.

#### 2.2.2. Surface Functionalization

Surface functionalization strategies involve the selective modification of the outermost layer of dental materials, enabling the introduction of bioactive properties while preserving the structural integrity and mechanical performance of the bulk material [88]. In contrast to bulk incorporation, surface-based approaches decouple biological functionality from the load-bearing core, making them particularly suitable for restorations, implants, and prosthetic components exposed to complex oral environments [88].

**Figure 6:**
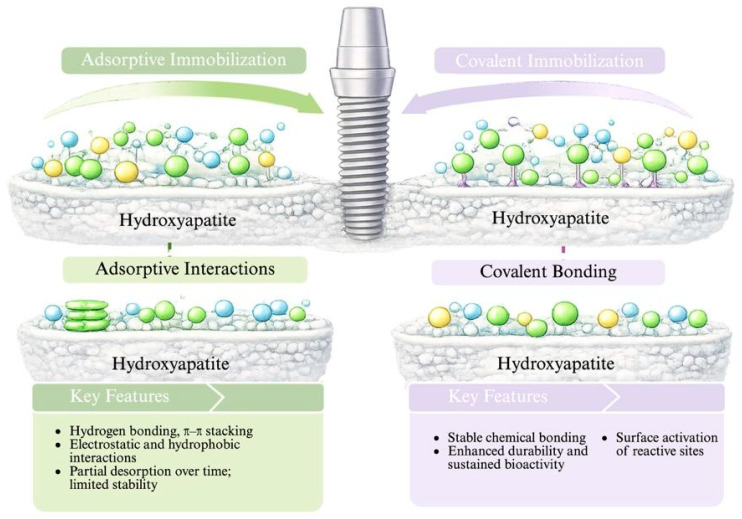
Schematic representation of adsorptive and covalent immobilization of bioactive compounds on hydroxyapatite surfaces used in dental materials and implant coatings.

From a mechanistic perspective, surface functionalization relies on physicochemical or covalent interactions between natural compounds and reactive groups present at the material surface [89]. Depending on the substrate composition and surface pre-treatment, natural compounds may be immobilized through adsorptive interactions, chemical grafting, or intermediate coupling layers [90]. Adsorptive immobilization is typically governed by hydrogen bonding, electrostatic interactions, hydrophobic forces, or π–π stacking, resulting in a reversible attachment that allows partial desorption over time. While this approach is relatively simple, it may lead to limited durability under mechanical stress, salivary flow, or chemical challenges [91]. Figure 6 illustrates the two main approaches for immobilizing bioactive compounds on hydroxyapatite surfaces: adsorptive immobilization and covalent immobilization. Adsorptive immobilization is based on weak interactions such as hydrogen bonding, electrostatic interactions, and hydrophobic interactions, which allow the attachment of bioactive molecules to the surface without chemical modification. However, this method may result in partial desorption over time and limited long-term stability.

In contrast, covalent surface functionalization provides enhanced stability by forming chemical bonds between functional groups on the natural compound (e.g., hydroxyl, carboxyl, amine groups) and activated surface moieties. Surface activation techniques—such as plasma treatment, silanization, or chemical etching—are commonly employed to introduce reactive sites, thereby promoting stronger and more durable anchoring [85]. This covalent immobilization restricts molecular mobility at the interface, ensuring sustained surface bioactivity while minimizing uncontrolled release into the surrounding environment [92].

Surface functionalization also directly influences interfacial phenomena, including protein adsorption, bacterial adhesion, and host tissue integration. By altering surface energy, wettability, and charge distribution, immobilized natural compounds can modulate early biological responses without compromising bulk mechanical behavior [93]. Importantly, because the modification is confined to a shallow surface layer, the risk of altering polymer network architecture, phase stability, or crosslinking density within the core material is substantially reduced [94,95].

Compared with bulk incorporation, surface functionalization offers superior control over biological activity while preserving bulk mechanical integrity, making it particularly advantageous for load-bearing dental applications where interfacial phenomena dominate clinical performance.

#### 2.2.3. Coating and Layered Systems

Coating and layered systems involve the deposition of a discrete bioactive layer containing natural compounds onto the surface of preformed dental materials, thereby creating a functionally stratified architecture in which mechanical support and biological activity are spatially separated [96]. Unlike bulk incorporation or surface functionalization, coating strategies introduce an additional material phase, whose properties and stability critically determine clinical performance [96].

From a mechanistic perspective, coating depositions may be achieved through physical attachment, chemical bonding, or hybrid approaches that combine both mechanisms [97]. Physically deposited coatings rely on weak interfacial forces, such as van der Waals interactions or mechanical interlocking, and are therefore more susceptible to delamination under cyclic loading, thermal fluctuations, and chemical challenges present in the oral environment [97]. In contrast, chemically bonded coatings involve covalent or coordination bonds formed between the coating layer and the underlying substrate, providing enhanced adhesion strength and improved resistance to mechanical and hydrolytic degradation [96,97].

Layered systems enable high local concentrations of natural compounds at the material–tissue interface, maximizing biological efficacy while avoiding perturbation of the bulk material’s polymer network or crystalline structure [98]. However, the introduction of a distinct coating layer also creates a critical interfacial zone, were mismatches in elastic modulus, thermal expansion, or surface energy may generate residual stresses. These stresses can promote crack initiation, coating fatigue, or gradual loss of bioactive material during long-term service [96,97,98].

Coating integrity and thickness uniformity are additional mechanistic determinants of performance. Excessively thin coatings may undergo rapid depletion or incomplete surface coverage, whereas thicker layers can compromise adhesion and increase the risk of cohesive failure within the coating itself. Moreover, coatings containing natural compounds often exhibit time-dependent degradation or release, governed by diffusion, dissolution, or enzymatic processes, which must be carefully balanced to avoid premature loss of function or uncontrolled exposure [99].

#### 2.2.4. Hybrid Natural–Synthetic Material Systems

Hybrid natural–synthetic material systems are based on the deliberate integration of bioactive natural compounds within engineered synthetic carriers or scaffolds, forming composite architectures that enhance stability, handling, and functional predictability [95]. Rather than introducing natural compounds directly into dental materials, hybrid systems employ an intermediate structural phase that governs compound distribution, retention, and interaction with the host matrix.

From a mechanistic standpoint, hybridization addresses key limitations associated with natural compounds, including chemical instability, limited solubility, volatility, and susceptibility to degradation during processing [100]. Synthetic carriers—such as polymeric microstructures, inorganic frameworks, or composite scaffolds—act as protective domains that physically confine natural molecules and modulate their exposure to the surrounding material environment. This confinement reduces premature loss or inactivation while enabling controlled integration into complex dental material systems [101].

The interaction between natural compounds and hybrid carriers is governed by physicochemical compatibility, including polarity matching, functional group complementarity, and interfacial energy balance. These interactions influence not only compound retention but also the overall behavior of the composite during fabrication. For example, hybrid carriers may alter rheological properties, curing kinetics, or phase organization, thereby affecting processing windows and manufacturability. When appropriately designed, hybrid systems can preserve structural continuity and mechanical integrity while incorporating bioactivity in a predictable manner [101,102].

However, hybrid material systems introduce additional interfaces and hierarchical complexity, which can influence long-term performance. The stability of carrier–matrix interactions becomes a critical determinant of durability, as interfacial debonding, carrier aggregation, or uneven dispersion may lead to localized heterogeneities and stress concentration sites [103]. Furthermore, reproducibility and scalability present nontrivial challenges, as hybrid formulations require precise control over component ratios, dispersion methods, and processing conditions.

To facilitate a clear material-centered comparison, the principal strategies employed for incorporating natural compounds into dental materials are comparatively summarized in Table 1, with emphasis on compound localization, interaction mechanisms with the host material, effects on bulk properties, and key advantages and limitations relevant to long-term clinical performance.

### 2.3. Impact of Natural Compounds on Dental Material Properties

**Figure 7:**
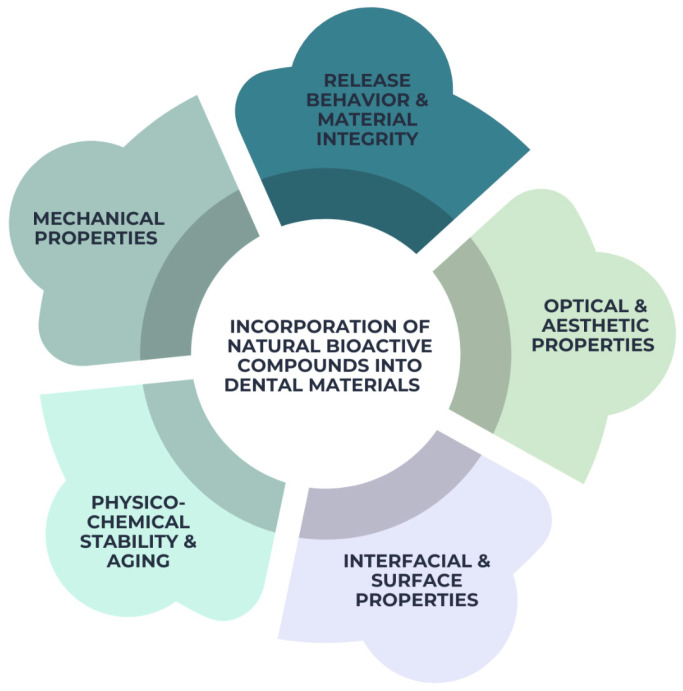
Main categories of properties influenced by the incorporation of natural bioactive compounds into dental materials, including mechanical properties, release behavior and material integrity, optical and aesthetic properties, interfacial and surface properties, and physicochemical stability and aging.

The integration of natural compounds into dental materials can affect many physicochemical and mechanical qualities, depending upon the class of compound, its concentration, and the method of incorporation [104]. From a materials science standpoint, these effects must be rigorously assessed to guarantee that functional improvements do not undermine structural integrity or long-term performance. Figure 7 illustrates the main categories of properties influenced by the incorporation of natural bioactive compounds into dental materials. Mechanical properties include parameters such as flexural strength, hardness, and elastic modulus, which determine the structural performance of dental materials. Release behavior and material integrity refer to the release of bioactive compounds over time and their influence on the structural stability of the material. Optical and aesthetic properties include color stability, translucency, and gloss, which are important for restorative materials. Interfacial and surface properties include surface roughness, adhesion, wettability, and biofilm interaction. Physicochemical stability and aging refer to water sorption, solubility, degradation, and long-term material stability in the oral environment.

#### 2.3.1. Mechanical Properties

Natural compounds can affect mechanical parameters such as flexural strength, elastic modulus, fracture toughness, and wear resistance. In polymer-based dental materials, the presence of low-molecular-weight natural compounds may interfere with polymer chain packing or crosslinking, potentially leading to reduced mechanical strength when incorporated beyond optimal concentrations [105]. Conversely, certain macromolecular compounds, particularly polysaccharides or protein-derived components, may contribute to reinforcement or energy dissipation when adequately integrated. The net mechanical outcome is therefore highly formulation-dependent and underscores the importance of concentration control and material compatibility [106].

In addition to intrinsic material composition, the mechanical response of dental polymers containing natural compounds is strongly influenced by compound dispersion and microstructural homogeneity. Uniformly distributed additives may promote more effective stress transfer, whereas aggregation or phase separation can generate stress concentrators that facilitate crack initiation and propagation.

Beyond static mechanical parameters, natural compounds may also alter viscoelastic behavior, including creep, stress relaxation, and fatigue resistance, which are critical for long-term performance under cyclic oral loading [104]. Furthermore, modifications in surface energy and hydrophilicity may increase water sorption and hydrothermal plasticization, exacerbating mechanical degradation in the oral environment [107]. These observations highlight an inherent trade-off between biological functionality and mechanical reliability, emphasizing the need for precise concentration thresholds and material–compound compatibility.

Experimental studies have demonstrated that the incorporation of chitosan into resin-based dental composites can significantly alter flexural strength and elastic modulus, with mechanical performance strongly dependent on chitosan concentration and its compatibility with the polymer matrix [108,109].

Comparative evaluations of bioactive restorative materials have revealed differences in flexural strength, elastic modulus, and fracture behavior when compared with conventional resin composites, highlighting the mechanical trade-offs associated with the introduction of functional additives [110,111]. The addition of natural bioactive compounds to conventional glass ionomer cements has been reported to provide limited or no improvement in mechanical properties, underscoring the material-dependent nature of mechanical reinforcement and the importance of matrix–additive compatibility [112,113].

#### 2.3.2. Physicochemical Stability and Aging

Physicochemical stability represents a critical determinant of clinical longevity. Natural compounds may alter water sorption, solubility, and hydrolytic stability of dental materials, particularly in hydrophilic systems [114]. Increased water uptake can accelerate aging processes, promote plasticization, and compromise interfacial bonding [104]. Additionally, susceptibility to thermal degradation, photooxidation, or enzymatic breakdown may influence long-term stability. Accelerated aging studies have highlighted the need to balance bioactive incorporation with resistance to environmental stressors present in the oral cavity [115,116].

Several studies have demonstrated that the incorporation of natural compounds into polymer-based dental materials can increase water sorption and solubility, particularly in hydrophilic matrices, thereby accelerating material aging and dimensional instability [117,118].

Increased water uptake associated with bioactive additives has been shown to promote hydrolytic plasticization of polymer networks, leading to reductions in stiffness, strength, and interfacial integrity over time [119,120]. Accelerated aging protocols have revealed that dental materials containing natural compounds may exhibit enhanced susceptibility to hydrothermal degradation, emphasizing the importance of evaluating long-term physicochemical stability under simulated oral conditions [121].

Thermal stability analyses indicate that certain natural compounds may lower the thermal resistance of polymer-based dental materials, potentially affecting performance during fabrication, sterilization, or prolonged intraoral temperature fluctuations [122].

#### 2.3.3. Optical and Aesthetic Properties

Optical characteristics, including color stability, translucency, and gloss retention, are particularly sensitive to the incorporation of natural compounds. Pigmented molecules, such as certain polyphenols or plant-derived extracts, may induce discoloration or color shifts over time, especially under light exposure. These effects are of particular relevance for aesthetic restorative materials, where even minor optical changes can impact clinical acceptability. Material formulations must therefore account for both initial appearance and long-term aesthetic stability [123].

Light exposure has been shown to exacerbate color changes in dental materials containing light-sensitive natural compounds, primarily through photooxidative reactions that alter chromophore structure and optical appearance [124].

Experimental investigations indicate that the addition of natural compounds may reduce material translucency by increasing light scattering within the polymer matrix, particularly when compound dispersion is non-uniform or when refractive index mismatch occurs [125].

Accelerated aging studies have demonstrated that optical changes, including discoloration and loss of translucency, may intensify following thermal cycling or UV exposure, underscoring the importance of long-term aesthetic assessment [122,123].

#### 2.3.4. Interfacial and Surface Properties

Surface characteristics, including roughness, surface energy, and wettability, may be modified by the presence of natural compounds, either through bulk incorporation or surface-specific strategies. Alterations in surface chemistry can influence adhesion to surrounding materials, interaction with oral fluids, and susceptibility to surface degradation. Maintaining controlled surface properties is essential to preserve material performance and ensure predictable behavior under functional loading

Experimental studies have shown that the incorporation of natural compounds into dental materials can modify surface roughness, either by altering polymerization behavior in bulk-modified systems or by inducing microstructural heterogeneity at the surface [120,124].

Several investigations have reported that natural compounds may significantly influence surface energy and wettability, particularly when polar functional groups are introduced, thereby affecting interactions with oral fluids and biological components [125].

Changes in surface chemistry induced by natural additives have been shown to affect adhesion to surrounding restorative or luting materials, potentially influencing interfacial strength and long-term bonding stability. Altered surface properties have been associated with increased susceptibility to surface degradation, including hydrolytic attack, wear, and chemical erosion, particularly under simulated oral aging conditions [126].

#### 2.3.5. Release Behavior and Material Integrity

Although the release of natural compounds from dental materials is often discussed in functional terms, from a materials perspective it primarily reflects matrix stability and structural coherence. Uncontrolled release may indicate inadequate compound retention or matrix incompatibility, leading to porosity, microcrack formation, or compromised mechanical performance. Controlled retention within the material framework is therefore critical to maintaining structural integrity over time [127]. Uncontrolled or accelerated release has been associated with inadequate compound retention and poor compatibility between the natural additive and the material matrix, often indicating structural instability at the microscale [12,128].

Experimental investigations have shown that excessive release of embedded compounds may promote porosity development and microstructural defects, which can act as initiation sites for crack formation [14,129].

The formation of microcracks linked to compound leaching has been reported to compromise mechanical performance, particularly under cyclic loading and long-term aging conditions [130]. Accelerated aging studies have correlated increased compound release with progressive matrix degradation, highlighting release behavior as an indirect marker of material durability [131].

While natural compounds are increasingly explored for their antimicrobial and bifunctional potential, their integration within dental materials may induce complex and sometimes adverse changes in mechanical, physicochemical, optical, and interfacial properties [132]. These modifications are often concentration-dependent and may reflect alterations in polymer crosslinking, filler–matrix interactions, and long-term stability under oral aging conditions.

The incorporation of natural bioactive compounds into dental materials may influence physicochemical, mechanical, and optical properties depending on the type and concentration of the compound. For example, chitosan incorporation has been reported to influence water sorption, mechanical strength, and degradation behavior of polymer-based materials [35,38,39]. Polyphenols and flavonoids may interact with polymer matrices and affect cross-linking density and mechanical properties [71]. Essential oils and propolis extracts may influence optical properties such as color stability and gloss due to their intrinsic pigmentation and interaction with the material matrix [74,75]. In restorative materials, color changes are often evaluated using ΔE values, while surface appearance may be assessed using gloss measurements. These changes are clinically relevant because they may influence restoration aesthetics, surface roughness, bacterial adhesion, and long-term material performance in the oral environment. Table 2 synthesizes the key material property domains influenced by natural compound incorporation and highlights the associated risks from a materials science standpoint.

### 2.4. Patents of Natural Compounds in Dentistry

In recent years, increasing attention has been given to the development of dental materials and therapeutic systems incorporating natural compounds, which has led to a growing number of patents in this field. These patents focus on the use of natural compounds due to their antimicrobial, anti-inflammatory, antioxidant, and regenerative properties, making them suitable for various dental applications [133].

According to the patent literature, the top pharmaceutical company using innovative technology to make vitamin C oral care products is Spoke Sciences, according to research in the Orbit Intelligence software licensed version v2.0.0 for patent database documents [134]. Several other pharmaceutical companies are also using similar technologies. Patent EP3445356 concerns a vitamin C herbal mixture and is owned by Spoke Sciences. Multiple advantages, including increased bioavailability, absorption, and a quicker start of action, are associated with this composition’s oral delivery strategy [134].

Additionally, it offers faster onset of action, larger peak concentrations, and improved therapeutic efficacy on both a subjective and objective level. The formulation of the herbal composition includes active ingredients like vitamin C, rutin, quercitrin, curcumin, resveratrol, limonene, and linalool; N-acylated fatty amino acids (linear, branched, cyclic, bicyclic, or aromatic); and plant extracts from various species.

Oral hygiene products are available in various forms, with mouthwashes representing one of the most common innovations in preventive dentistry. Some patented oral hygiene formulations incorporate natural bioactive compounds with antimicrobial and anti-inflammatory properties. For example, the patent KR102372384, entitled “Oral hygiene composition for preventing or alleviating periodontal disease and halitosis”, describes a mouthwash formulation containing natural compounds such as collagen, Ginkgo biloba extract, tocopherol (vitamin E), Allium sativum (garlic) extract, and vitamin C, along with other bioactive agents including lysozyme chloride and zinc compounds. These natural components contribute to antimicrobial activity, reduction in inflammation, and improvement of periodontal health, making the composition effective in preventing or alleviating periodontal disease and halitosis [134].

Even though dental implants are the standard of excellence for tooth loss due to periodontal disease, there are nevertheless common biological effects such as inflammation and bone loss around the implants. “Immunomodulatory, oral microbiome altering and tissue regenerative oral care compositions and methods of use in the prevention and treatment of periodontal and peri-implant diseases,” which is the title of patent US20230270761, describes the use of a therapeutic hydrogel in non-surgical ways to treat peri-implant diseases and aid in the healing of periodontal tissues [134].

The hydrogel composition incorporates a number of bioactive compounds derived from natural sources. Xylitol, vitamin C, peppermint oil, spearmint oil, tetrahydrocurcuminoids, myrrh oil, Chinese star anise extract, and Aloe vera powder are all part of this. Water and glycerin constitute the foundation of the recipe. Regenerative alterations include periodontal tissue healing, oral microbiota modulation, and peri-implant tissue health restoration [134]. helped by the anti-inflammatory, antibacterial, antioxidant, and regenerativ properties of these natural compounds

Among all compounds mentioned in patents, green tea ranked second. The development of technological items pertaining to green tea has made oral patches an important subject of study for patents [135]. The conventional mechanical treatment for periodontitis, which includes scaling and root planing, can be enhanced with the use of oral patches. One of the reasons this patch is so effective is that it keeps the medication where it was applied for a long time. As an example, a novel patch that can be fastened to a tooth or a peripheral part is described in patent document KR102309837. You can simply brush off this patch, and it will stay put. What stays is the support layer that contains the medicinal layer with at least one active ingredient, such as Sanguinarine or Triclosan. Herbal extracts from plants like pumpkin [134].

The increasing number of patents related to natural compounds in dentistry highlights the growing interest in bioactive and biomimetic dental materials. These developments demonstrate the translational potential of natural compounds from experimental research to clinical and commercial dental applications.

## 3. Future Directions and Limitations

Future research should prioritize the standardization and rational design of dental materials incorporating natural compounds. Establishing well-defined protocols for compound sourcing, characterization, and incorporation is essential to reduce variability and improve reproducibility across studies. Comparative investigations using standardized material formulations and testing conditions would enable more robust structure–property correlations.

The review relies on preclinical and laboratory-based evidence, with relatively limited long-term validation under clinically relevant conditions. Differences in material formulation, testing protocols, and aging simulations across studies further constrain the ability to generalize findings or to establish definitive structure–property relationships.

Further work is needed to clarify the long-term stability and aging behavior of natural compound–modified dental materials under clinically relevant conditions. Systematic aging studies addressing hydrolytic, thermal, and mechanical stressors would provide critical insight into durability and performance over time, which remains insufficiently documented in the current literature.

Advances in material engineering strategies, including controlled incorporation approaches and hybrid material systems, may help overcome existing limitations related to compatibility and stability. Future studies should focus on optimizing concentration thresholds and incorporation methods to balance functional enhancement with preservation of mechanical and optical properties. As this review focuses on material-level considerations, clinical performance and in vivo outcomes were not systematically assessed, and therefore translational implications should be interpreted with caution. Future systematic reviews and standardized experimental frameworks are needed to strengthen evidence integration and support clinical translation.

In addition, the development of standardized evaluation frameworks tailored to natural compound–based dental materials would facilitate cross-study comparability and translational assessment. Such frameworks should integrate physicochemical, mechanical, and surface-level analyses within a unified material science perspective.

Although many studies report positive effects of natural bioactive compounds on dental materials, the results are not always consistent across studies. The effect of natural compounds strongly depends on concentration, extraction method, chemical composition, and interaction with the material matrix. In some studies, the incorporation of natural compounds improved antibacterial properties but negatively affected mechanical strength, color stability, or polymerization behavior. Other studies reported limited long-term stability or rapid release of the bioactive compounds, reducing their long-term effectiveness. In addition, variability in natural extract composition and lack of standardization represent important limitations. Therefore, although natural bioactive compounds show promising potential, further studies are necessary to optimize concentration, incorporation methods, and long-term performance in dental materials.

Finally, while this review emphasizes material-level considerations, future interdisciplinary research bridging material science with preclinical validation may support the rational translation of these systems toward clinical relevance, provided that material performance remains the primary design driver.

Despite the promising biological activity of natural bioactive compounds, their incorporation into dental materials must be carefully optimized, as improvements in biological properties may be accompanied by changes in mechanical, optical, or physicochemical properties. Therefore, the development of multifunctional dental materials requires a balance between biological activity and material performance.

This review has several inherent limitations related to its narrative design. Although the literature search was broad and conceptually structured, it did not follow a fully systematic protocol, and therefore the selection of studies may be influenced by publication availability, indexing practices, and language restrictions. As a result, relevant negative findings or less frequently cited studies may be underrepresented.

Another important limitation arises from the heterogeneity of the included literature. The analyzed studies span diverse experimental designs, material systems, compound classes, and evaluation methodologies, which limit direct comparability across studies and precludes quantitative synthesis. Consequently, the conclusions drawn are primarily qualitative and integrative in nature.

A further limitation of this review is the non-systematic inclusion of grey literature. Non–peer-reviewed sources, such as technical reports, theses, conference proceedings, and preprints, were not systematically searched or included. While this approach may exclude some emerging or preliminary data, it was intentionally adopted to prioritize peer-reviewed, methodologically transparent evidence and to ensure consistency and quality of the analyzed material science data. Consequently, the findings reflect established trends within the indexed literature rather than exhaustive evidence mapping.

The translation of dental materials containing natural bioactive compounds from laboratory research to clinical application requires compliance with regulatory frameworks and performance standards. Dental materials must meet requirements related to biocompatibility, mechanical performance, stability, and safety before clinical use. Regulatory approval typically requires evaluation of cytotoxicity, biocompatibility, degradation behavior, and long-term stability. In addition, materials must demonstrate adequate mechanical properties, color stability, wear resistance, and durability in the oral environment. Therefore, the incorporation of natural bioactive compounds must not compromise the fundamental performance requirements of dental materials. Future research should focus not only on biological activity but also on regulatory compliance, standardization of natural extracts, long-term stability, and clinical performance evaluation.

Although natural bioactive compounds show promising biological properties, their successful clinical implementation depends on regulatory approval, material standardization, long-term stability, and the preservation of essential mechanical and optical properties required for dental materials.

## 4. Conclusions

This narrative review highlights the expanding interest in incorporating bioactive natural compounds into contemporary dental materials and underscores the complexity of translating biological functionality into reliable material systems.

Natural bioactive compounds have emerged as promising functional components for the development of advanced dental materials. Their incorporation into dental materials can be achieved through multiple strategies such as bulk modification, surface functionalization, coating systems, and encapsulation approaches, depending on the material type and desired release behavior.

The available literature shows that natural bioactive compounds can enhance antimicrobial activity, surface bioactivity, and interaction with biological tissues, but they may also influence mechanical, physicochemical, optical, surface, and release-related properties of dental materials. Therefore, the integration of natural compounds into dental materials requires a balanced approach that considers both biological functionality and material performance.

Future research should focus on controlled incorporation strategies, nanostructured delivery systems, long-term material stability, standardization of natural compounds, and clinical validation to ensure their safe and effective implementation in dental materials.

## Figures and Tables

**Table 1:** Comparative overview of material-based strategies for incorporating natural compounds into dental materials.

Strategy	Localization of Natural Compounds	Interaction Mechanism with Material	Effect on Bulk Properties	Control of Bioactivity	Main Advantages	Main Limitations
Bulk incorporation [81,82,83]	Distributed throughout the entire material volume	Physicochemical interactions with monomers and polymer chains (hydrogen bonding, van der Waals, π–π interactions)	High—may affect polymerization kinetics, crosslinking density, phase stability, and mechanical properties	Low to moderate	Simple formulation; homogeneous volumetric distribution	Risk of plasticization, phase separation, reduced mechanical strength; limited biological efficiency of embedded compounds
Surface functionalization [88,89,90]	Confined to the outermost surface layer	Adsorptive or covalent bonding to activated surface functional groups	Minimal—bulk structure remains unaffected	High	Preserves mechanical integrity; precise control of interfacial bioactivity	Limited modification depth; potential loss of activity if surface bonds are unstable
Coating and layered systems [92,93,94,95]	Discrete bioactive layer on preformed material	Physical adhesion, chemical bonding, or hybrid interfacial mechanisms	None to minimal	High, but time-dependent	High local bioactive concentration; spatial separation of structure and function	Adhesion failure, coating fatigue, interfacial stress, durability issues under oral conditions
Hybrid natural–synthetic systems [100,101,102,103]	Confined within synthetic carriers integrated into the material	Carrier-mediated stabilization and interfacial interactions	Variable—dependent on carrier–matrix compatibility	High and tunable	Improved compound stability; controlled distribution; customizable material design	Increased formulation complexity; reproducibility and scalability challenges

**Table 2:** Impact of Natural Compounds on Dental Material Properties.

Material Property Domain	Potential Impact of Natural Compounds	Material Science Considerations
Mechanical properties [104,105,106,107,108,109]	Alteration of flexural strength, elastic modulus, fracture toughness, and wear resistance	Concentration-dependent effects; possible interference with polymer crosslinking or reinforcement effects
Physicochemical stability [114,115,116,117,118,119,120]	Changes in water sorption, solubility, and aging behavior	Risk of plasticization, hydrolytic degradation, and reduced long-term durability
Optical and aesthetic properties [122,123]	Color shifts, discoloration, reduced translucency or gloss stability	Pigmented compounds and photo-instability may compromise aesthetic longevity
Surface and interfacial properties [120,124,126]	Modification of surface roughness, wettability, and surface energy	Influences adhesion, surface degradation, and interaction with oral fluids
Releasing behavior and integrity [127,128,129]	Potential uncontrolled release or leaching of compounds	Indicative of matrix incompatibility, porosity, or compromised structural coherence

## Data Availability

No new data were created or analyzed in this study. Data supporting the findings of this review are available within the cited literature.

## Abbreviations

The following abbreviations are used in this manuscript:

DD%	Degree Of Deacetylation
NPs	Native Peptides
HA	Hydroxyapatite
UV	Ultraviolet Radiation
ΔE	Color Difference
